# Supplementary material for: Combined Cytotoxic Effect of Inhibitors of Proteostasis on Human Colon Cancer Cells
Source: Pharmaceuticals (Basel). 2022 Jul 25;15(8):923. doi: 10.3390/ph15080923 (PMC9331496; doi:10.3390/ph15080923)
Supplement: Supplementary file 1 [file pharmaceuticals-15-00923-s001.zip › pharmaceuticals-1752348-supplementary.pdf]

pharmaceuticals

MDPI

Supplementary Material

to m/s

Combined Cytotoxic Effect of Inhibitors of Proteostasis on Human Colon Cancer Cells

Alina D. Nikotina, Snezhana A. Vladimirova, Nadezhda E. Kokoreva, Elena Y. Komarova, Nikolay D. Aksenov, Sergey Efremov, Elizaveta Leonova, Rostislav Pavlov, Viktor G. Kartsev, Zhang Zhichao, Boris A. Margulis and Irina V. Guzhova

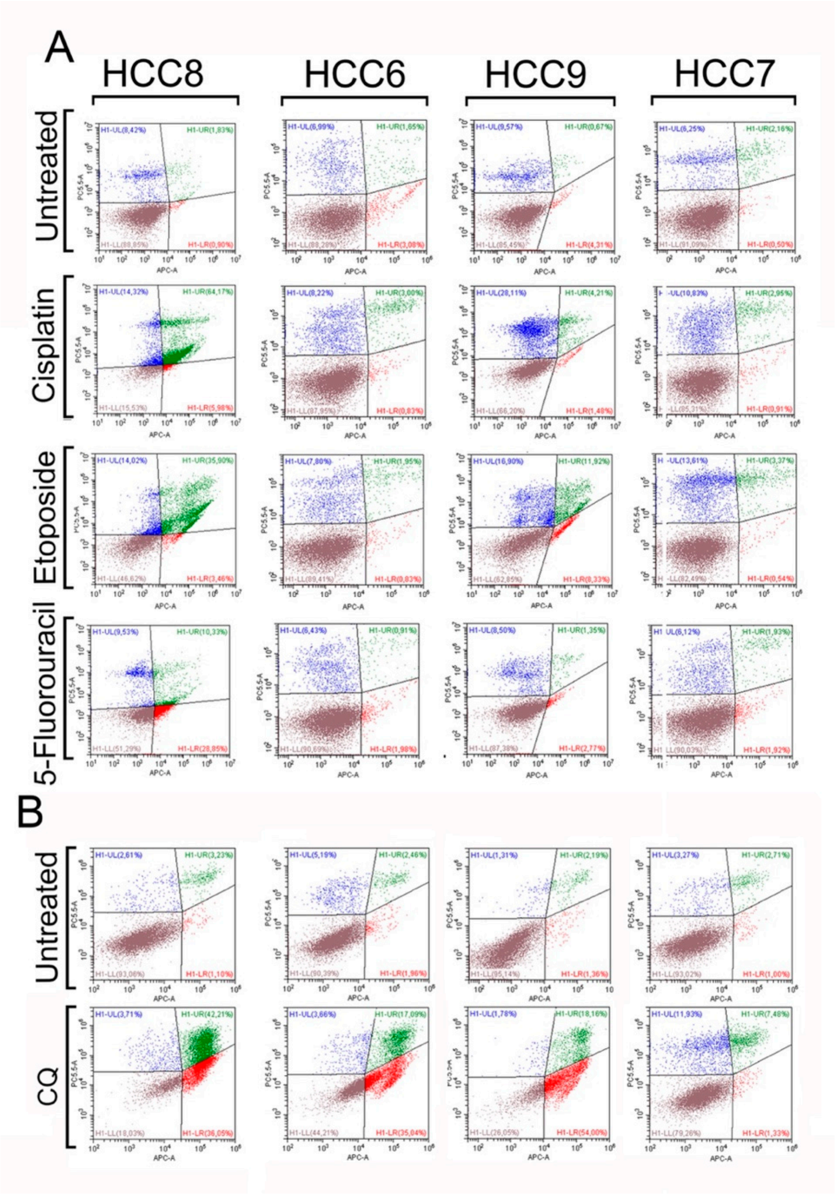

Figure S1. Flow cytometry diagrams presented on Figure 1D in main text

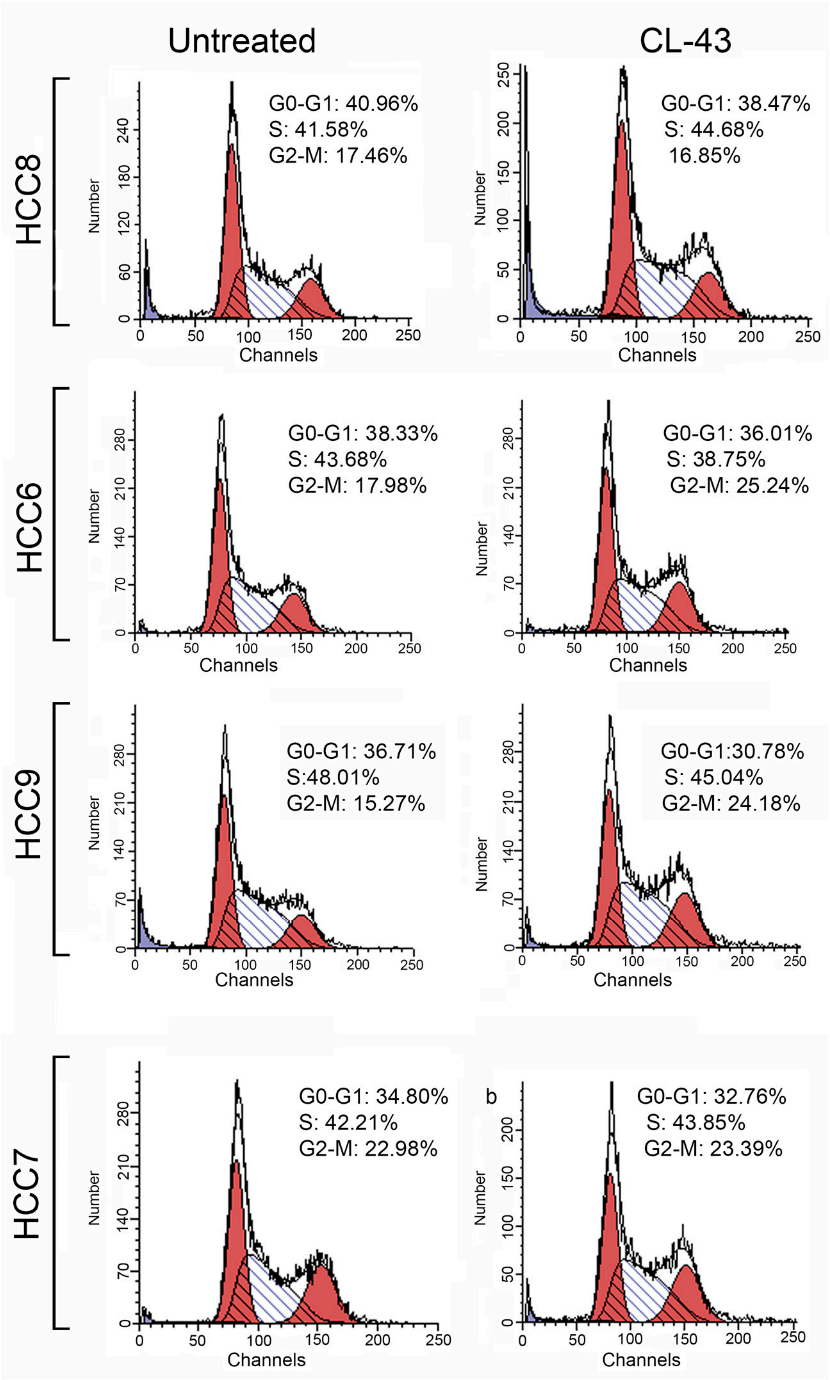

Figure S2. Cell cycle analysis of HCC cells after treatment with 500 nM of CL-43

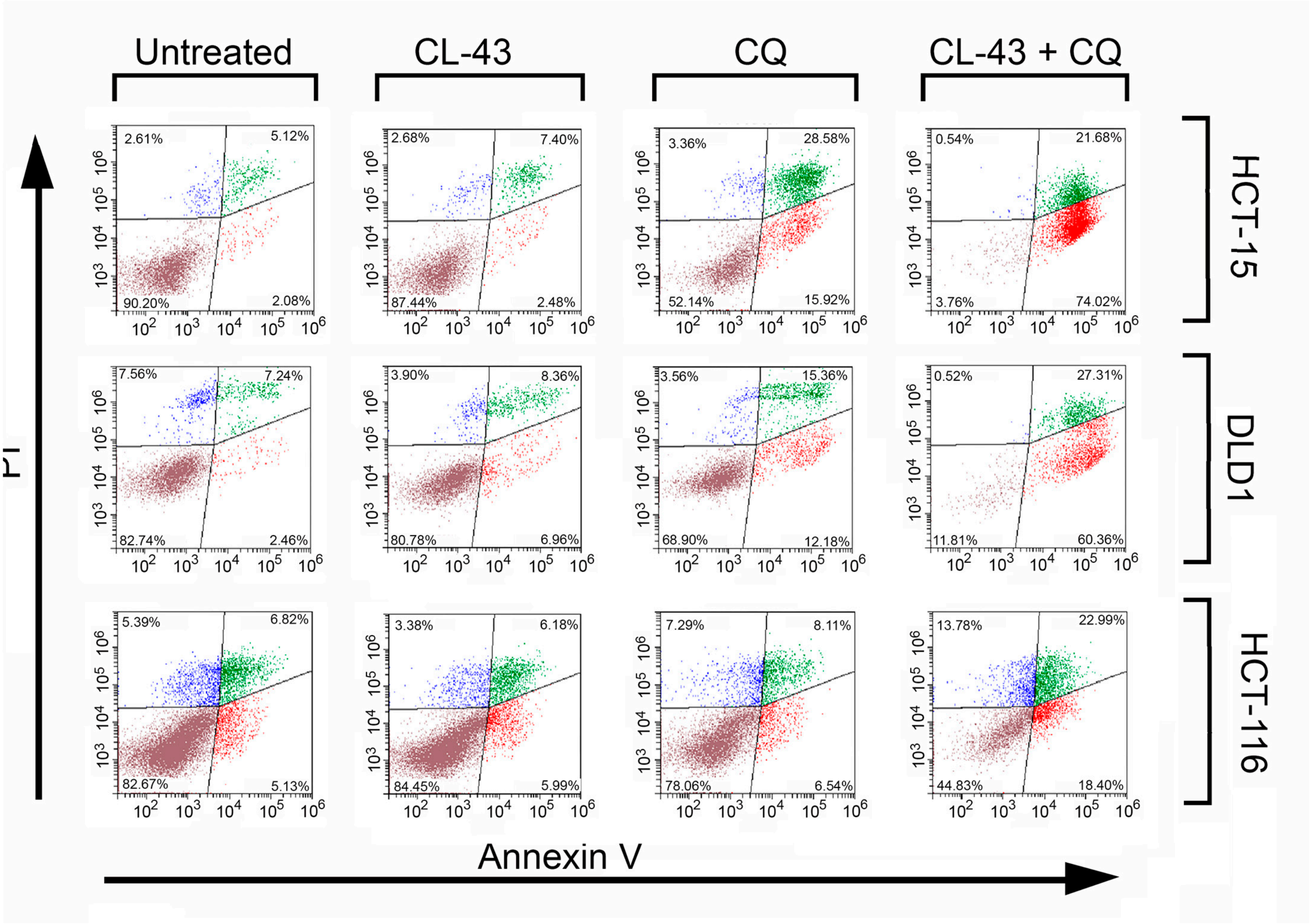

Figure S3. Flow cytometry diagrams presented on Figure 4D in main text
